# Unveiling origins, composition, and appearance of ancient Islamic gold coins through elemental and smartphone-based colorimetric studies

**DOI:** 10.1038/s41598-024-53981-6

**Published:** 2024-02-13

**Authors:** Roberto Sáez-Hernández, María Josefa Luque, Adela R. Mauri-Aucejo, Ángel Morales-Rubio, M. Luisa Cervera

**Affiliations:** 1 Department of Analytical Chemistry, Faculty of Chemistry, University of Valencia, Research Building, C/ Dr. Moliner, 50, 46100 Burjassot, Spain; 2 Department of Optics and Optometry and Vision Sciences, Faculty of Physics, University of Valencia, C/ Dr. Moliner, 50, 46100 Burjassot, Spain

**Keywords:** Imaging studies, Analytical chemistry, Sensors

## Abstract

In this article, the Islamic gold coins collections of the University of Valencia is studied for the first time for its elemental composition and colorimetric properties. To that end, non-destructive elemental analysis using energy-dispersive X-ray fluorescence is applied to obtain the coins’ elemental profile. Additionally, the colour of the coins is assessed using smartphone-based colorimetry as an innovative non-invasive method. Results indicate that the Islamic coins could be attributed to Almoravids, and the gold origin could be the famous Sudanese gold, an ore which was valued all over the world. Also, the text found in the coins was translated and allowed to objectively identify the mint and year. Based on these results, it can be seen that the earliest coins struck in the Iberian Peninsula are characterised by slightly lower gold concentrations than the ones struck in the northern part of Africa, pointing towards a potential recycling of coins which already circulated in the area. In conclusion, this work provides new analytical insights into a peculiar and unique type of samples, allowing to draw some conclusions in terms of their origins and materials, and for the first time allows to characterise the chromatic coordinates of this type of samples.

## Introduction

The Iberian Peninsula has been home to different civilizations and cultures. Among them, the Islamic presence from the year 711 to 1492 of the Common Era (CE) is of special significance, given its wide time span (more than 700 years in total). As a consequence, numerous cultural patterns and assets can be traced back to their culture, including art, architecture, language and literature. Among the rests that can be still found in archaeological findings are coins. These are a very special type of sample, more so if gold coins are considered, given the fact that their corrosion is very limited, and hence they can be conserved in very good conditions, unlike other types of organic or inorganic samples which degrade or corrode over time.

An area of special interest is the analytical investigations of historical artefacts, given that variations of their major elements can be a sign of economic wealth or constraints, while their trace constituents can be often used as a chemical marker in order to identify the origin of the raw material and/or the manufacturing process.

Given the intrinsic cultural, economic, and historical value of the samples under investigation, chemical analysis and approaches are limited to non-destructive and non-invasive techniques. The most commonly used techniques in this field are X-ray fluorescence (XRF), proton induced X-ray emission (PIXE), muonic X-ray emission spectroscopy (μXES) or Laser Ablation Inductively Coupled Plasma Mass Spectrometry (LA-ICP-MS). Each one of these techniques present different advantages and drawbacks, based on the cost, sensitivity, representativity and availability.

Among them, XRF is the frequently used given its simplicity, relative low cost and availability. These characteristics have turned this technique into a reference approach for gold and silver-based jewellery^1^. Multiple examples of its application to the elemental study of historical coins are available. For example, the analysis of Etruscan gold coins^2^, coins minted in the Italic Peninsula between 12 and 14 centuries^3^, or a variety of Roman coins^4^. However, its use involves some limitations: first, the sensitivity of the instrument is not great for some elements, and hence the presence of some interesting elements which are precious for provenance studies might not be above the limits of detection. Second, the instrument only interacts with the very first microns of the samples^5^, and hence elemental information is only representative for the outermost layer of the coin. In this sense, other more sophisticated techniques like muonic X-ray emission spectroscopy (μXES) allow to study the elemental composition through different points within the core of the coin^6^.

LA-ICP-MS application to coin analysis allows to carry out provenance studies since it can differentiate between different isotopes^7^, and it has been used to analyse the provenance of Celtic coins^8^ and to study different types of gold coins in the Iberian Peninsula^9^. However, this technique is slightly invasive, since it requires to minimally alter the sample. PIXE analysis is also present in the literature^10^, for example, in the study of gold artifacts from Xiongnu^11^, and the assessment of gold and metallic objects from the Visigoths^12^.

As said, all this information about an archaeological sample is often used to study the provenance of the materials used during the minting. However, there are two difficulties in this approach. First, it must be considered that minting processes often require elevated temperatures, and sometimes purification processes are used. This means that some of the most distinctive elements can be evaporated from the sample, leaving an information hole behind. Second, most of the natural sources of the ores have run out nowadays, and so a direct comparison between the original material and the sample is sometimes not possible^13^.

One of the most characteristic aspects of gold coinage is their eye-catching look. This is reflected on the colour of the samples, which will depend upon the metallic elements that compose their most outer layer. To the best of the authors’ knowledge, no analytical investigation on the colour of gold coins is present in the literature. A new insight into the chromatic characteristics of the Islamic Almoravid dinars is then presented in this work, using a smartphone-based procedure developed to tackle two of the most significant distortion factors in the image capture process (specular reflection and the influence of lighting conditions).

This work first focuses on the elemental composition of the dinar collection of the University of Valencia, based on XRF analysis, in order to shine light into the provenance of the coins, and to assess the differences between different geographical and time origins. At a second stage, a chromatic study of the samples is carried out to characterise the colour of these samples for the first time in this context.

## Objective

The objective of this work is to assess, for the first time, a significant fraction of Islamic gold dinars belonging to the Historical Library of the University of Valencia, and to study the chromatic coordinates of the samples using smartphone-based colorimetry.

## Materials and methods

### Samples’ description

The analysis has been carried out on 58 gold coins (Table S1) belonging to the Historical Library of the University of Valencia. Figure S1 shows pictures of some representative coins. The physical properties (mass and size) as well as a brief description of the coins are shown in Table S1. The size of the coins was measured using two perpendicular measurements, given that their contours were not perfectly round. On each face, inscriptions are found indicating the year and place of minting. The text stamped in both faces was translated and used to identify the mint, year, and authority at the time. Given the historical and economical value of these samples, the analysis was performed under some restrictions. First, the analysis had to be carried out in situ, since the coins could not leave the controlled environment of the library. Second, only non-destructive and non-invasive analysis methods were allowed.

### Portable X-ray fluorescence (pXRF)

A portable energy-dispersive X-ray fluorescence (p-ED-XRF) spectrometer Titan S1 from Bruker was used to measure major and minor elements in the sample, using the “Restricted materials” internal calibration and its application for metals from the manufacturer. Measurements were taken on both faces of the coins, and a minimum of 2 measurements per face were obtained: to that end, the coin was measured, turned 180° on the same face, measured again, and then flipped to repeat the process on the other face. The result for each coin was obtained by averaging the 4 measurements. At this stage it is important to stress out the impossibility of intervening in any way on the coins: these were only touched with cotton gloves, and no cleaning on the surface was allowed.

### Smartphone-based colorimetry

#### Instrumental setup for smartphone-imaging of ancient coins

The main challenge at this stage is the fact that historical gold coins are generally shinny and hence their colour characterization is complicated. In this work, the colour of the samples was measured by the means of a Samsung Galaxy Edge S7 model SM-G93F, with a 12.2 MP camera sensor. Images of both sides of the coin were captured, processed to obtain CIELAB values and averaged. The coins were placed on a white flat surface, placed within a white foam sphere with a hole on top, whose external faces were painted in black in order to avoid stray light. A white LED stripe on the inner diameter of the sphere, parallel to the ground, was used as a homogenous light source. The white surface at the base of the coin allowed for subsequent image cropping and served as a white reference for the computation of L*, a* and b* values. The smartphone was placed on an external horizontal support, with the main camera pointing down through the hole on the top of the sphere, and the built-in camera app was used in its automatic mode. With this set-up (see Fig. S2a), homogeneous illumination on the surface of the sample was accomplished, avoiding differences in illumination, glare or reflections of the coin that might interfere with the final result.

#### Image cropping and colour descriptors extraction

Images were stored in .*jpg* format and further transferred to Matlab to carry out image analysis using Matlab toolbox COLORLAB^14^, where a lab-made routine was built in order to extract RGB parameters from the surface of the coins. At this stage, the objective is to first separate the region of interest from the background, and then to extract their RGB values to be subsequently converted into CIELAB descriptors.

The image segmentation consisted of a two-step procedure: first, Matlab’s *rgb2lab* function was used, transforming the sRGB values^15^ to the CIELAB space. This yields a first estimation of the L*a*b* descriptors of the image pixels under the D65 illuminant, though not being exactly accurate in terms of colour reproduction, allows for a fast image segmentation. Next, K-means clustering was used to classify pixel colour into two groups (K = 2) based on lightness (L*), and chroma (C*-the modulus of the (a*,b*) vector). The region with the highest chroma and lightness belongs to the coin. This is schematically represented in Fig. S2b.

Since the coins presented some dirty or degraded parts, especially in their rougher regions, K-means was again used (with K = 2) to remove the darker pixels from within the coins, leaving those pixels that represent only the colour of the metal itself.

#### Smartphone characterisation

The colorimetric profile of the particular smartphone camera used was derived to avoid the drawbacks of using a generic colorimetric profile. To this purpose, a set of Munsell chips (sheets from 1.25G to 10YR, Munsell Book of Colour, Glossy Finish Collection, 1976, Macbeth, Kollmorgen Corporation, Baltimore, Maryland), covering the same region of CIELAB colour space than the coins, were photographed with the camera, and their reflectance spectra retrieved from the COLORLAB Munsell database^14^. These spectra were used to compute the reference CIELAB values for these chips. With these reflectances and the RGB values of the chips, a model was built. It consisted of a 5th degree polynomial that uses raw RGB data as input, and outputs CIELAB descriptors. The coefficients of this model were obtained by least squares minimization between the computed and the reference CIELAB descriptors of the Munsell chips.

Given that each photograph was taken in the automatic mode of the camera, slight changes among pictures are expected. To take that into account, Lab values are computed for both the sample of interest and a white stimulus illuminated as the samples (in the case of the coins, the white background). These values are converted to CIEXYZ values, using a reference illuminant (D65). Finally, new corrected Lab values are computed using the white sample in the scene as reference background. All this is summarised in Fig. S2c. Furthermore, the coins were measured with a reference spectroradiometer (SpectraScan PR650 from Jadak) using the same setup in order to evaluate the suitability of the colour estimation procedure.

### Statistical treatment

Data treatment and visualization was carried out using R^16^, and packages *ggplot2*^17^, *missMDA*^18^ and *corrplot*^19^. For Principal Component Analysis (PCA), data was mean-centred and scaled by their variance. Elements that were above the Limits of Detection (LOD) were selected.

## Results and discussion

### Physical description

Based on the information shown in Table S1, the average (n = 58) mass of the coin is 4.078 ± 0.020 g (confidence interval of the mean at 95%, CI95), while their diameter (obtained as the average value of two perpendicular measurements) is 25.25 ± 0.17 mm (CI95). These results provide and insight into the fineness of the minting process that the Almoravids had developed. Samples, as observed in Table S1, do come from different mints, both in the Northern part of Africa and the southern shore of the Iberian Peninsula, but their structure is very homogeneous, with dispersion values (expressed as the coefficient of variation) of 1.9 and 3.0% for the mass and size, respectively.

### Elemental composition

**Figure 1:**
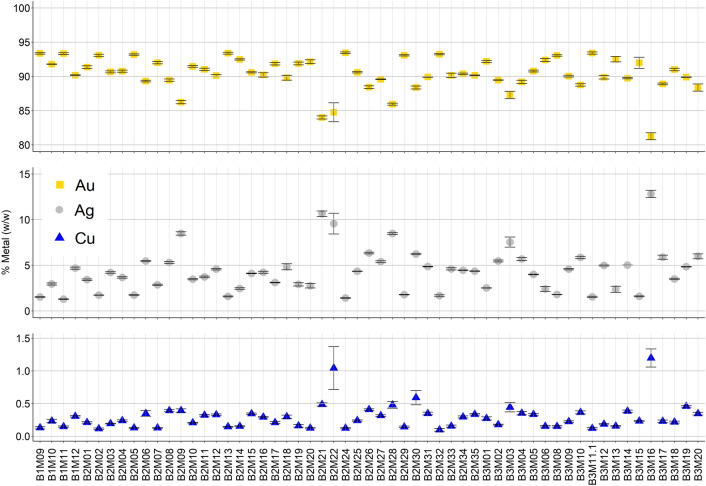
Elemental composition for all the coins under investigation. Results are expressed as mean and standard deviation.

The elemental composition is assessed using non-destructive portable energy dispersive X-ray fluorescence (p-ED-XRF). In Fig. 1 the three main constituents—Au, Ag and Cu—are plotted, while the detailed concentrations of these elements are summarised in Table S2. An example of the spectra is depicted in Fig. S3.

Some interesting remarks can be made from this information: first, the major element (considered as any element above 1%) is Au, whose distribution is concentrated around 90% Au or above (90.4 ± 2.5% w/w, mean and standard deviation). Next, as another major element Ag is found at smaller values of 4.4 ± 2.4% (w/w, average and standard deviation). Last, copper is only found at minor levels (0.1% to 1%) for most of the coins.

The distribution of the three elements is also interesting, since it allows to understand the dataset with more detail. The histograms for Au, Ag and Cu are plotted in Fig. S4. As observed, the most significant part of the distribution of the major element, Au, is found at values above 90% (w/w), finding only a few coins below 85%. Also, the Cu distribution is found at values below 1.5% in good accordance with the previous literature^20^.

**Figure 2:**
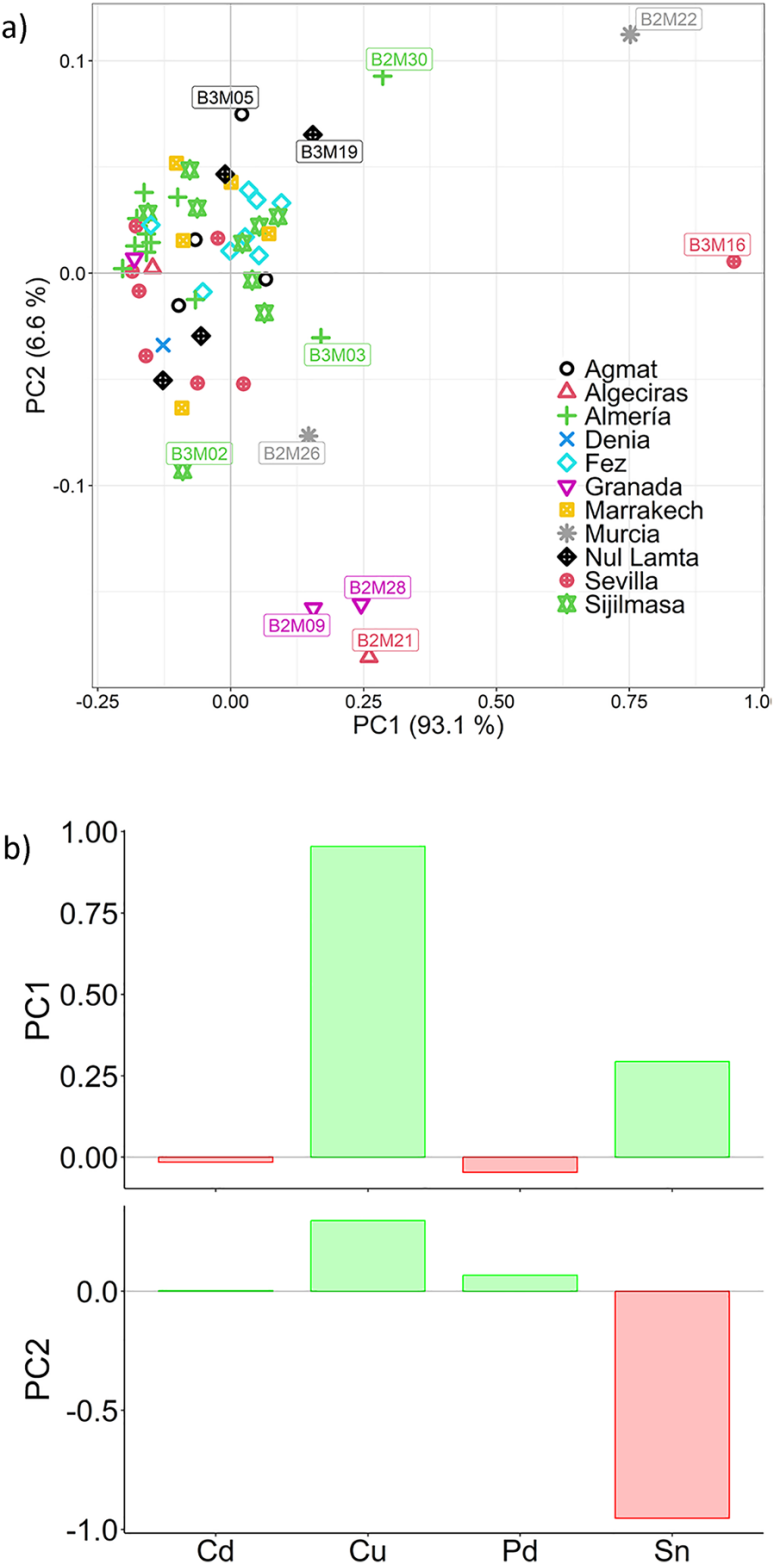
PCA results for the trace elements present in the samples. (**a)** Scores plot; (**b**) original variable—principal component correlation plot.

In order to assess the contribution of the trace elements, a Principal Component Analysis (PCA) is carried out excluding Au and Ag during the model creation, and using Cd, Cu, Pd and Sn as they are commonly studied in the investigation of trace elements in ancient coins^3,7,9,10^. The resulting scores and variables plots are presented in Fig. 2.

Principal Component (PC) 1 explains 93.1% of the total variance of the dataset, while PC2 explains 6.6%. It is seen in Fig. 2b that Cu and Sn are the two minor elements which generate differences on the data: Cu contributes positively to both PC1 and PC2, while Sn is negatively correlated to PC2, and presents a small positive contribution (0.29) to PC1. The PCA did not yield any specific grouping or clustering, pointing to a very homogeneous dataset in general, except for some samples which showed differential composition (B2M22, B3M16, B2M28, B2M09, B2M21) Interestingly, all these coins belong to Iberian mints, a result that will be further discussed next.

Samples B2M22 (Murcia, 509HY) and B3M16 (Sevilla, 504HY) are found at high levels of PC1, pointing to the presence of high amounts of Cu. On the other hand, B2M22, B3M05, B3M11 and B3M19 are found at high PC2, pointing to lower Sn contents. Contrarily, B2M09, B2M21, B2M26, B2M28, B2M33, B3M02, B3M03, and B3M20 are on the negative side of PC2, indicating high levels of tin.

This kind of analysis is often done when assessing historical metallic samples in order to compare their composition, since much of the information regarding the origin of the raw materials is not available: first, because the original mints are exhausted, and hence finding recent samples is almost impossible^13,21^; and second, because some elements were lost and/or modified upon their treatment at high temperatures (during amalgamation and refining, for instance)^21^.

**Figure 3:**
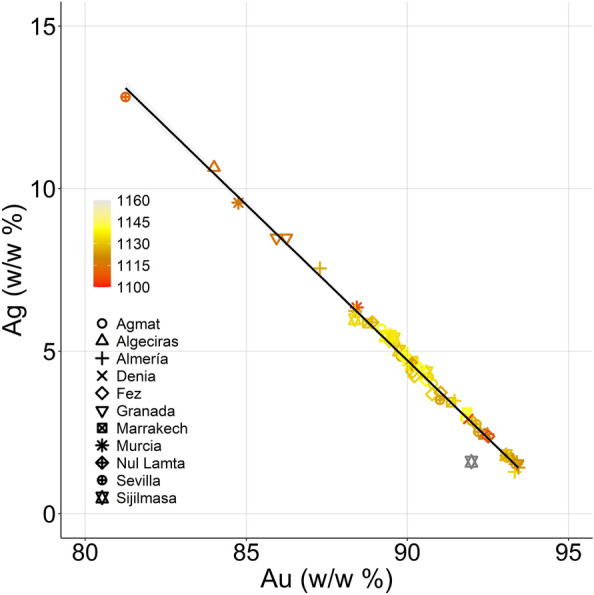
Relationship between Au and Ag content in the Islamic coins. Colour gradient depends on the year that is assigned to the coins following the information available on the library (Gregorian calendar).

The analysis of the Islamic coins in terms of their Au and Ag relationship reveals an almost perfect linear trend (R^2^ = 0.99, Fig. 3) with a negative correlation.

Figure 3 reveals that the coins with the smallest amount of gold are attributable to the first years of the investigated period (marked in orange). This, however, is not always the case, as there are some other coins from that same period presenting high gold concentrations. This result might point out that the year is not the only variable which influences the composition.

In this regard, it can be observed that coins with lower Au amounts are all assigned to mints founded within the Iberian Peninsula (Algeciras, Murcia, Granada, Almería), whilst coins with stable and more elevated levels of Au come from African mints (Fez, Marrakech, Sijilmasa, Nul Lamta). This same result has been previously mentioned in the literature^22^. Hence, linking this geographical aspect to the fact that coins with lower Au percentages belong to the earliest ones (Fig. 3), it could be hypothesized that a certain degree of recycling of coins with lower Au percentages which were already circulating in the Peninsula was taking place^13^.

Searching the literature for analysis of similar samples, it is found that a 92% of gold in dinars of this time has been previously described^23^, while some other works present values of 94% for Almoravid coins^13^. Analysis on different dinars minted in different regions of North Africa and the Mediterranean by the year 1050–1100 report differences depending on the area: for instance, Messier states that dinars from Fatimid Egypt contained around 97.4% Au, while eastern regions had around 95.6%. This author, regarding the Almoravid dinars, reports a 92% value^20^. Almoravid coins from this time, according to the literature, were found around 92.2% Au^23^, which is in very good accordance to the results presented in this work. Overall, these results are summarized in Table 1.

**Table 1:** Comparison of the Au contents as described in the literature and the ones described in this work.

Sample	Au content (w/w) percentage (n = number of samples)*	References
Dinars from Fatimid Egypt	97.4	Messier^20^
Dinars	95.6	Messier^20^
Almoravid dinars	94* (n = 8)	Gondonneau and Guerra^13^
Almoravid dinars	92.2	Hébert^23^
Almoravid dinars	92	Messier^20^
Almoravid dinars	90.4 ± 2.5 (n = 57)	This work

*n* number of samples, when disclosed.

*This value has been calculated by the authors based on the data available in the article.

From Table 1, it can be seen that the data presented in this work is accordant to the data already available in the literature. Specially, it becomes interesting the work of Gondonneau and Guerra^13^ as they present the data for 8 Almoravid coins with a very similar distribution as the one we observed in Fig. S4. Also, it must be considered that our dataset is significantly bigger than the one presented in many of the referenced works, so the influence of coins with low Au contents, as observed in Fig. 1, is affecting the final mean value.

In any case, what is observed in our dataset is that the coins assessed in this work contain a very homogeneous and elevated level of Au, which is characteristic of the Almoravid period^22^. As can be seen in Fig. 3, and as was mentioned previously on the text, values of 90.4 ± 2.5% w/w Au are found in the samples under investigation This result is in almost perfect correlation to the previous reports from Messier^20^. Nonetheless, some coins are found at significantly lower values, around 82–87%.

Reports about potential debasement of the coins have been previously published elsewhere. For instance, Gondonneau and Guerra^13^ published that major debasement was observed on Abbasid coins found in North Africa and Egypt. These authors also reported that the decrease in Au content was correlated to the increase in Ag while keeping Cu at very low levels (< 1%) due to the non-purification of the gold ore. Additionally, a second possibility can be considered: that Au was debased by adding Ag on purpose. This would imply, given the fact that Ag was usually obtained from Pb mines, that a positive correlation between Ag and Pb would be found^13^. In the context of this study, Pb was not found above the limits of detection of the instrument, so further insights in this direction are not possible.

Following previous analysis, it has been suggested that Almoravids used the famed “Sudanese gold”, which arrived to north-western parts of Africa and the Iberian Peninsula through gold caravan routes^13,21^. This is the same material that merchants around the world valued as very pure and valuable^20,23^. Consequently, this would explain the very homogeneous results in terms of composition found in our dataset, since exploring it through ratios of elements like Pd, Pt, Cu and Ag did not yield any specific grouping apart from the detection of some differential coins.

**Figure 4:**
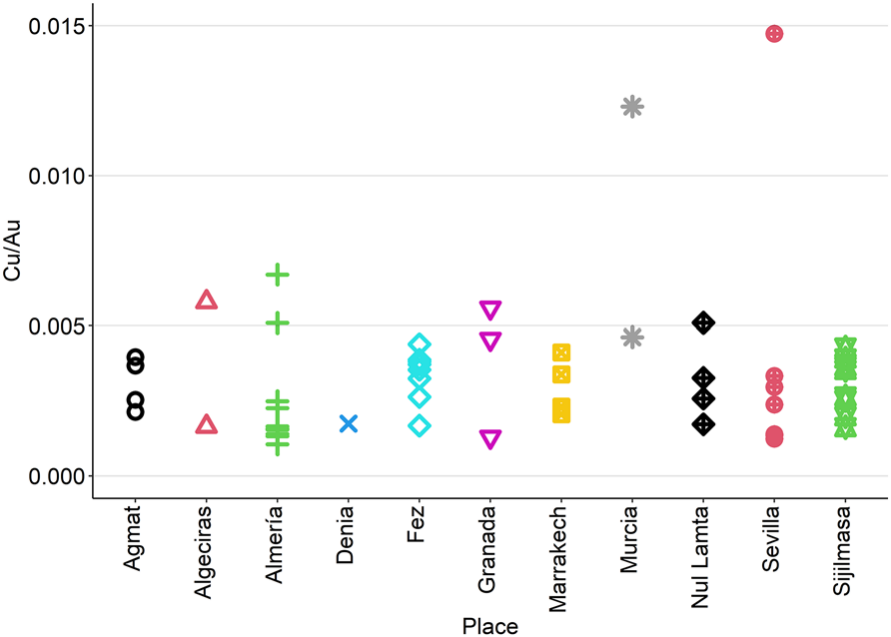
Cu/Au relationship for the Islamic coins depending on their minting place.

The presence of Cu in the samples has been previously used to compare coins produced in different cities and those from Sijilmasa, considered as reference^20^. This is done because it is considered that every coin minted in Sijilmasa by Almoravids was made from Sudanese gold, given the close proximity and the geographical control that this people had on the region. The use of Cu as a tracing element is based on the fact that it is usually found naturally along with Au in proportions from 1 to 5%, approximately. This content varies depending on the origin of the ore, so it is a marker for differential origins^24^. Applying that methodology to the dataset under investigation (Fig. 4), it can be seen that the majority of the samples (except for B3M16 and B2M22, which already appeared as outliers in Figs. 2, 3) are similar to the ones from Sijilmasa. Hence, this is further evidence for their homogeneity in terms of origin of their gold.

As Messier wrote^20^, and as has been previously developed in the text, the Almoravids were renowned as merchants all over the Mediterranean area, and the reputation of their coins was based on the provenance of their gold from the valued Western mines. Hence, they did not find any reason to further refine the Au from their ore, and struck coins using the raw material as it was found. This would explain the perfect correlation found between Au and Ag in the coins under investigation. The fact that Sudanese gold found nowadays in that same area has been proven to contain 92% Au^20^, along with the evidence shown so far, could potentially point towards the use of this raw material in these coins.

### Colorimetric analysis of the samples

#### Validation of the smartphone-based procedure

First, the quality of the colour measurement is assessed. The results obtained through the procedure described in “Smartphone-based colorimetry” section yielded a series of colour descriptors, which can be compared to the reference values obtained through the spectrocolorimeter.

**Figure 5:**
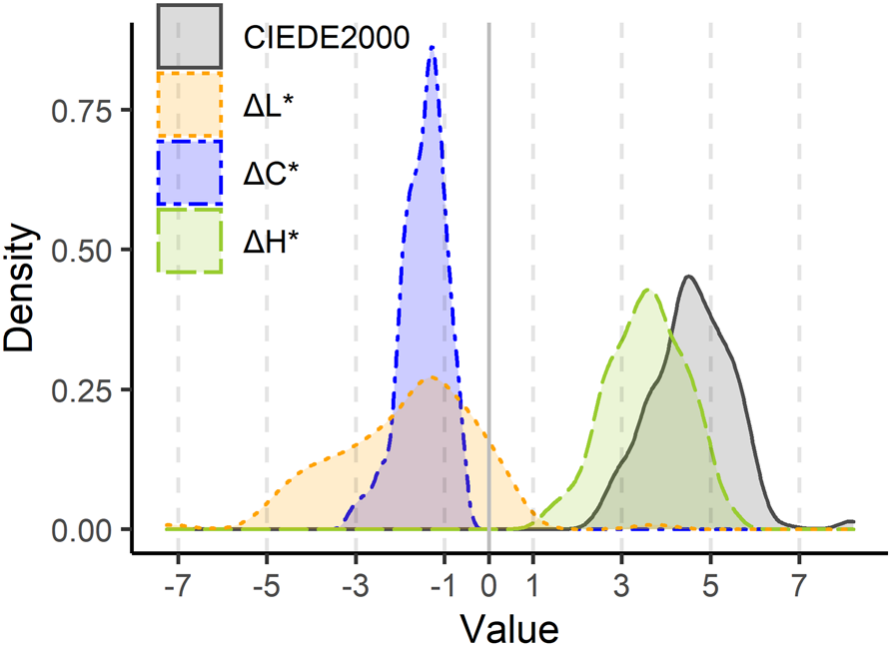
Density plot of the distance between the reference values and the corrected values through smartphone-based imaging. ΔL*, ΔC* and ΔH* are used to calculate CIEDE2000, and all four parameters are displayed.

In Fig. 5, the distance between the corrected image-based parameters and the reference values are plotted using the CIEDE2000 distance. This metric calculates the difference between two colour descriptors using ΔL*, ΔC* and ΔH* (change in lightness, chroma and hue, respectively) and is usually used in colorimetry to assess the degree of similarity between two colour measurements^25,26^. Its interpretation is as follows: as the CIEDE2000 metric is closer to 0, both measurements are more alike. Hence, if the density plot of the hole sample population is plotted, an overall estimation of the quality of colour reproducibility can be obtained.

As can be observed, DE2000 values present a non-normal distribution (Shapiro–Wilk *p*-value = 0.000049) with a median value of 4.6 units and a IQR (Inter Quartile Range) of 1.2. This value is slightly higher than the stablished threshold of 3.5, which is set in the literature as the limit to consider two colours as indistinguishable in printed samples^25^. However, each component building up to the CIEDE2000 value can be studied separately. That is, CIEDE2000 is constructed on the basis of ΔL*, ΔC* and ΔH* (expressed as reference − corrected image value), which account for the difference in lightness (L*), chroma (C*) and hue (h*) in the CIELCh colour space^26^.

Individually assessing each one of them, it is observed that ΔH* (change in hue) is the one that deviates more from 0, with a normal distribution (Shapiro–Wilk *p*-value = 0.08) centred at 3.5 ± 0.9 (mean and standard deviation). This parameter takes positive values, meaning that the smartphone is capturing slightly lower hue values than the reference device. As per ΔC*, it presents a median value of − 1.4 (IQR 0.7), being the most similar parameter captured between both devices. This result means that the smartphone captures slightly more colourful colour than the reference device. Lastly, ΔL* (median − 1.6, IQR = 2.2) is mainly plotted taking negative values, pointing to a more lightful colour captured by the smartphone. The differences in ΔC* and ΔL* together might be explained by the fact that, during the image processing steps, the dirty parts of the coins were discarded.

In this regard, it must be taken into consideration that these comparisons between both devices were made despite not sampling the exact same spot. That is: while the reference device only registers a circle which is centred on the centre of the coin, the colour represented by the smartphone belongs to the area extracted during the image treatment steps (as described in “Smartphone-based colorimetry” section). This means that the image-based protocol does only contain information about the clean parts, unlike the reference values. Hence, despite the slight differences in the sample regions, having colour differences values (expressed as CIEDE2000) around 4.6 units can be considered accurate.

The smartphone approach to the analysis of historical coins’ colour presents some major drawbacks. First, as mentioned above, this method yields information only about the outermost layer of the sample. However, this problem does not relate to the analytical tool itself, the smartphone, but rather to the colorimetry investigation. Thus, the results must be considered taking this into account. Regarding the analytical implementation of smartphones, it should be considered that they present two major sources of error: on the one hand, the fact that they are not originally designed as analytical tools. In this sense, manufacturers often implement automatic software that modifies and enhances colour. On the other hand, the results are very sensitive to lighting environment and changes. These are the reasons behind the need of implementing a characterisation approach, like the one presented here. As results demonstrate, the proposed procedure correctly tackles with these problems. The characterization only needs to be carried out once, so that the time investment becomes negligible if many samples are to be analysed. Lastly, it must be considered that the characterisation models are built to a specific device. This means that each correction model is built based on the properties of the smartphone that has been used. Hence, they cannot be applied if the images are taken with other devices.

But this approach also has potential advantages when compared to the reference one. For example, the possibility to analyse only representative fractions of the coins. These kinds of samples often come with some degraded or corroded parts which alter their final aspect. This means that a bulk analysis of the sample (like the one carried out by the reference device) can sometimes contain distorted information, while a more precise and versatile analysis can be made with the image-based procedure. Also, these devices are more widely present and available among the analysts’ community and are much more user-friendly. Thus, this smartphone-based approximation can help in describing these kinds of objects without the need of bulky and extensive equipment.

#### CIELAB descriptors of the samples

**Figure 6:**
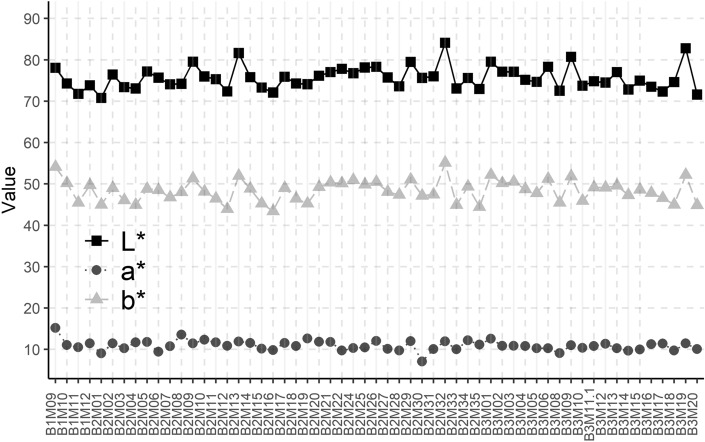
CIELAB descriptors for the coins under investigation, obtained through the proposed image-based procedure.

Based on the discussion above, the image-based CIELAB descriptors of the samples are analysed. In Fig. 6 the resulting CIELAB descriptors of the samples are represented.

It can be observed that the homogeneity among the samples is significant, with the following values, expressed as the mean and the standard deviation: L* = 76 ± 3; a* = 10.9 ± 1.2; b* = 48.4 ± 2.6. The extremely constant elemental composition of the coins, along with their physical shape and size, regardless of their year or city of origin, are also reflected on their colorimetric appearance. A correlation analysis between the colorimetric data and the elemental composition, more specifically the Au content, was investigated and no significant correlation was observed. This, rather than meaning that the elemental composition does not have an effect on the colour, is due to the nature of the dataset: in this case, the samples under investigation were Almoravid dinars, and as has been seen in Fig. 1, their elemental composition is very homogeneous. Thus, the final colour of the samples is also very homogenous, not allowing to build any significant correlation.

These results allow, for the first time, to have a quantitative description of Almoravid gold coinage. This parameter is often overlooked in the investigation of ancient coinage, and when mentioned, it is only assessed from a qualitative point of view^27,28^. Among the scarce literature on the topic, a quantitative description of two roman coins is reported using a colorimeter^29^. Comparing these results with the ones presented in this article, the L* of gold coins is slightly higher, while both a* and b* parameters are significantly higher for gold coins, especially the b* value, reflecting the yellow component of gold coins.

The investigation of colour of ancient coinage presents as an analytical approach with high potential, as cases of the application of colour to classify ancient coins has been reported elsewhere^30^, demonstrating its usefulness in the field. Thus, the methodological developments and validation presented here propose a new smartphone-based approach, with corrected CIELAB descriptors that accurately represent the colour of the samples. Also, the results shown in Fig. 6 quantitatively represent, for the first time, the colorimetric description of Almoravid dinars.

## Conclusions

In this work, the dinar coins collection of the University of Valencia has been analytically studied from the point of view of their elemental composition using portable X-ray fluorescence spectrometry. Results have demonstrated that the coins present a very homogeneous composition regardless of the origin, with average gold contents of 90.4 ± 2.5% (weight percentage). A more precise analysis allowed to find that some coins struck in the southern regions of the Iberian Peninsula presented lower gold contents, with a significant and negative correlation with silver content. This result could point to a certain degree of reutilization of already present coins in these regions during the first years of the Islamic presence in the Peninsula. Additionally, comparison among the coins with the ones originating from Sijilmasa allowed to see that their composition was, in most of the cases, similar. On the basis of this fact, the provenance of the material could be related to the renown Sudanese gold.

References to the colour and appearance of historical coins are common in the specialized literature. However, the lack of objective values and descriptions of the samples burden the analytical approach. In this context, it becomes evident that the colour of these archaeological artifacts is a parameter of interest. The most straightforward analytical approach to capture colour is through colorimeters. These, despite yielding colour descriptions of the samples, work by averaging a specific region of the coin with a bulk approach. When dealing with historical coins, usually containing dirty of corroded areas, these methods fall short as they do not allow to capture the colour of the metal on the surface.

To that end, in this work a novel analytical approach to study the colour of gold coins, using a smartphone colorimetrically characterised to work as a colorimeter is proposed and validated. The comparison between the image processing-based method and the values obtained through the reference device demonstrated that the proposed setup works satisfactorily for this analytical purpose. To the further of our knowledge, this is the first time a non-invasive method to characterize the colour of historical gold coins is proposed in the literature. By the implementation of the proposed method, a very fast and easy way of selectively capture the colour of historical coins is presented. Based on these results, preliminary conclusions about the potential composition can be obtained to build a more sophisticated approach upon them. Also, this data can help classifying and sorting the different coins based on objective parameters rather than subjective criteria.

All in all, this work shines a light on a significant collection of valuable gold coins, which enrich the knowledge about Almoravid gold coins and their presence in the Iberian Peninsula, and proposes a new analytical method to widen the information available about historical gold coins.

### Supplementary Information

Supplementary Information.

## Data Availability

Data is available using a link within the supplementary information file.

## Abbreviations

BCE: Before common era
CE: Common era
CI95: Confidence interval 95%
HY: Hegira year
IQR: Inter quartile range
LA-ICP-MS: Laser ablation inductively coupled plasma mass spectrometry
PC: Principal component
PCA: Principal component analysis
p-ED-XRF: Portable energy dispersive X-ray fluorescence
PIXE: Proton induced X-ray emission
