# Supplementary material for: Unveiling origins, composition, and appearance of ancient Islamic gold coins through elemental and smartphone-based colorimetric studies
Source: Sci Rep. 2024 Feb 13;14:3652. doi: 10.1038/s41598-024-53981-6 (PMC10864403; doi:10.1038/s41598-024-53981-6)
Supplement: Supplementary file 1 — Supplementary Information. [file 41598_2024_53981_MOESM1_ESM.pdf]

SUPPLEMENTARY INFORMATION

for

Unveiling origins, composition, and appearance of ancient Islamic gold coins through elemental and smartphone-based colorimetric studies

Roberto Sáez-Hernández[1]*, María Josefa Luque[2], Adela R. Mauri-Aucejo[1], Ángel Morales-Rubio[1], M. Luisa Cervera[1]*

[1]Department of Analytical Chemistry, Faculty of Chemistry, University of Valencia. C/ Dr. Moliner, 50. Burjassot, 46100, Spain.

[2]Department of Optics and Optometry and Vision Sciences, Faculty of Physics, University of Valencia, Burjassot, Spain. C/ Dr. Moliner, 50. Burjassot, 46100, Spain.

Corresponding authors: Roberto Sáez-Hernández and M. Luisa Cervera

Phone number: +34 963543486 (shared)
Email: roberto.saez@uv.es and m.luisa.cervera@uv.es
Address: Research Building – Department of Analytical Chemistry, Faculty of Chemistry, University of Valencia. C/ Dr. Moliner, 50. Burjassot, 46100, Spain.

Table S1: Sample's physical and historical description. The year is expressed as the Islamic calendar.

Sample	Mass (g)	Diameter (mm)	Width (mm)	Description (as obtained in the translation)	Year	Place	Origin	Vives reference
**B1M09**	3.968	24.6 - 24.9	0.6 - 0.9	Ali Ibn Yusuf	515	Almería	Islamic	1646
**B1M10**	4.138	25.5 - 26.0	0.6 - 0.8	Ali Ibn Yusuf, El Amir Sir	532	Almería	Islamic	1726
**B1M11**	4.177	25.7	0.6 - 0.9	Ali Ibn Yusuf, El Amir Sir	528	Almería	Islamic	1747
**B1M12**	4.149	25.8 - 26.2	0.6 - 0.7	Ali Ibn Yúsuf, El Amir Sir	527	Marrakech	Islamic	1738
**B2M01**	4.148	25.8 - 24.8	0.5 - 0.6	Ali Ben Yúsuf, El Amir Sir	524	Marrakech	Islamic	1736
**B2M02**	3.972	24.7 - 24.8	0.6 - 0.7	Alí Ben Yúsuf	516	Sevilla	Islamic	1657
**B2M03**	4.138	25.9	0.4 - 0.6	Ali Ben Yúsuf, El Amir Sir	529	Agmat	Islamic	1723
**B2M04**	4.160	25.2 - 25.5	0.5 - 0.7		527	Fez	Islamic	1758
**B2M05**	3.925	25.0 - 25.2	0.5 - 0.7	Ali Ben Yúsuf, El Amir Sir	518	Sevilla	Islamic	1660
**B2M06**	4.144	25.8 - 26.2	0.6 - 0.8	Alí Ibn Yúsuf	531	Sijilmasa	Islamic	1720
**B2M07**	4.041	24.5 - 24.8	0.5 - 0.8	Ali Ben Yúsuf, El Amir Sir	521	Sevilla	Islamic	1665
**B2M08**	4.140	25.5 - 26.0	0.5 - 0.7	Ali Ibn Yúsuf	530	Fez	Islamic	1761
**B2M09**	3.976	23.8 - 24.2	0.7 - 0.8	Ali Ben Yúsuf, El Amir Sir	509	Granada	Islamic	1610
**B2M10**	4.169	25.9 - 26.1	0.5 - 0.7	Ali Ibn Yúsuf	527	Almería	Islamic	1746
**B2M11**	4.053	25	0.5 - 0.7	Ali Ben Yúsuf, El Amir Sir	521	Fez	Islamic	1976
**B2M12**	4.135	25.1 - 25.5	0.5 - 0.7	Ali Ibn Yúsuf	530	Sijilmasa	Islamic	1719
**B2M13**	3.978	25.4 - 25.6	0.5 - 0.7	Ali Ben Yúsuf, El Amir Sir	519	Almería	Islamic	1645
**B2M14**	4.172	25.2 - 25.3	0.6	Alí Ibn Yúsuf	514	Almería	Islamic	1753
**B2M15**	4.169	26.4 - 26.5	0.6	Ali Ben Yúsuf, El Amir Sir	534	Fez	Islamic	1784
**B2M16**	4.110	25.3 - 25.4	0.6 - 0.7	Ali Ben Yúsuf, El Amir Tashfine	527	Fez	Islamic	1798
**B2M17**	4.158	25.66 - 25.7	0.5 - 0.6	Ali Ben Yúsuf, El Amir Sir	535	Marrakech	Islamic	1803
**B2M18**	4.005	24.7 - 25.1	0.5 - 0.7	Ali Ben Yúsuf, El Amir Tashfine	520	Sevilla	Islamic	1663
**B2M19**	4.078	24.7 - 25.1	0.5 - 0.7	Ali Ibn Yúsuf	499	Denia	Islamic	1522
**B2M20**	4.054	24.7 - 24.8	0.6 - 0.7	Emir Yusuf Ibn, Emir Ali	521	Sevilla	Islamic	1665
**B2M21**	4.043	23.7 - 23.8	0.6 - 0.8	Alí Ibn Yúsuf	507	Algeciras	Islamic	1634
**B2M22**	3.944	23.7 -23.8	0.6 - 0.8	Ali ben Yúsuf	509	Murcia	Islamic	1632
**B2M24**	3.981	24.6 - 24.8	0.6 - 0.7	Alí Ibn Yúsuf	518	Almería	Islamic	1649
**B2M25**	4.135	25.8 - 25.9	0.5 - 0.6	Alí Ibn Yúsuf	528	Sijilmasa	Islamic	1731
**B2M26**	4.030	24.0 - 24.2	0.6 - 0.7	Ali Ben Yúsuf, El Amir Sir	501	Murcia	Islamic	1624
**B2M27**	4.113	25.1 - 25.6	0.5 - 0.7	Alí Ibn Yúsuf	529	Sijilmasa	Islamic	1718
**B2M28**	3.992	23.5 - 23.6	0.6 - 0.7	Ali Ben Yúsuf El Amir Sir	509	Granada	Islamic	1610
**B2M29**	3.958	24.5 - 24.7	0.5 - 0.6	Alí Ibn Yúsuf	515	Almería	Islamic	1646
**B2M30**	4.118	24.9 - 25.6	0.5 - 0.6	Alí Ibn Yúsuf	522	Almería	Islamic	1653
**B2M31**	4.153	25.1 - 25.3	0.5 - 0.6	Alí Ibn Yúsuf	531	Fez	Islamic	1762
**B2M32**	3.982	24.8 - 25.1	0.6 - 0.7	Ali Ben Yúsuf El Amir Sir	517	Almería	Islamic	1648
**B2M33**	4.120	26.1 - 26.3	0.5 - 0.6	Alí Ibn Yúsuf	518	Nul Lamta	Islamic	1981
**B2M34**	4.122	25.8 - 25.9	0.5 - 0.7	Alí Ibn Yúsuf	535	Nul Lamta	Islamic	1787
**B2M35**	4.138	25.3 - 25.7	0.5 - 0.7	Ali Ben Yúsuf, El Amir Tashfine	525	Fez	Islamic	1729

Table S1 (continued): Sample's physical and historical description. The year is expressed as the Islamic calendar.

Sample	Mass (g)	Diameter (mm)	Width (mm)	Description	Year	Place	Origin	Vives reference
**B3M01**	4.020	25.0 - 25.1	0.6 - 0.8	Alí Ibn Yúsuf	518	Sevilla	Islamic	1661
**B3M02**	4.125	26.6	0.4 - 0.7	Ali Ben Yúsuf, El Amir Sir	531	Sijilmasa	Islamic	1720
**B3M03**	3.982	25.4 - 25.5	0.5 - 0.8	Alí Ibn Yúsuf	520	Almería	Islamic	1691
**B3M04**	4.168	26.1 - 26.3	0.6 - 0.7	Alí Ibn Yúsuf	533	Agmat	Islamic	1727
**B3M05**	4.151	25.5 - 25.7	0.6 - 0.7	Ali Ben Yúsuf, El Amir Sir	529	Agmat	Islamic	1723
**B3M06**	3.952	24.6 - 25.0	0.5 - 0.8	Alí Ibn Yúsuf	510	Algeciras	Islamic	1641
**B3M08**	4.099	25.6 - 25.7	0.5 - 0.7	Ali Ben Yúsuf, El Amir Sir	523	Sijilmasa	Islamic	1713
**B3M09**	4.147	25.7 - 25.8	0.5 - 0.7	Ali Ben Yúsuf, El Amir Sir	531	Almeria	Islamic	1711
**B3M10**	4.117	25.7 - 25.8	0.5 - 0.7	Ali Ben Yúsuf, El Amir Sir	525	Marrakech	Islamic	1737
**B3M11.1**	3.965	24.3 - 24.9	0.6 - 0.8	Alí Ibn Yúsuf	515	Granada	Islamic	1654
**B3M11.2**	4.010	24.9 - 25.0	0.6 - 0.7	Alí Ibn Yúsuf	520	Almeria	Islamic	
**B3M12**	4.165	25.8 - 26.0	0.6 - 0.7	Ali Ben Yúsuf, El Amir Sir	539	Marrakech	Islamic	1577
**B3M13**	4.086	25.2 - 25.4	0.6 - 0.7	Alí Ibn Yúsuf	501	Fez	Islamic	1729
**B3M14**	4.150	25.0 - 25.1	0.6 - 0.8	Ali Ben Yúsuf, El Amir Sir	525	Sijilmasa	Islamic	1456
**B3M15**	4.008	24.4 - 24.7	0.6 - 0.7	Alí Ibn Yúsuf	-	Sijilmasa	Islamic	1991
**B3M16**	4.065	24.4 - 24.9	0.6 - 0.8	Alí Ibn Yúsuf	504	Sevilla	Islamic	
**B3M17**	4.136	24.4 - 24.9	0.6 - 0.7	Ali Ben Yúsuf, El Amir Sir	523	Nul Lamta	Islamic	1711
**B3M18**	3.935	25.1 - 25.5	0.6 - 0.9	Alí Ibn Yúsuf	519	Sevilla	Islamic	1662
**B3M19**	4.107	25.9 - 26.6	0.5 - 0.6	Ali Ben Yúsuf, El Amir Tashfine	535	Nul Lamta	Islamic	1787
**B3M20**	4.136	25.4 - 25.7	0.6 - 0.8	Ali Ben Yúsuf, El Amir Sir	528	Sijilmasa	Islamic	1717

Sample B3M07 was not available at the moment, so it could not be measured. Some of the coins were not present in the reference numismatic catalogue

Vives reference numismatic catalogue:

Antonio Vives y Escudero. *Monedas de las dinastías arábigo-españolas.* Establecimiento tipográfico de Fortanet, Madrid, 1893.

SOME OF THE COINS THAT WERE ANALYSED IN THIS WORK

a) **b)** **c)**

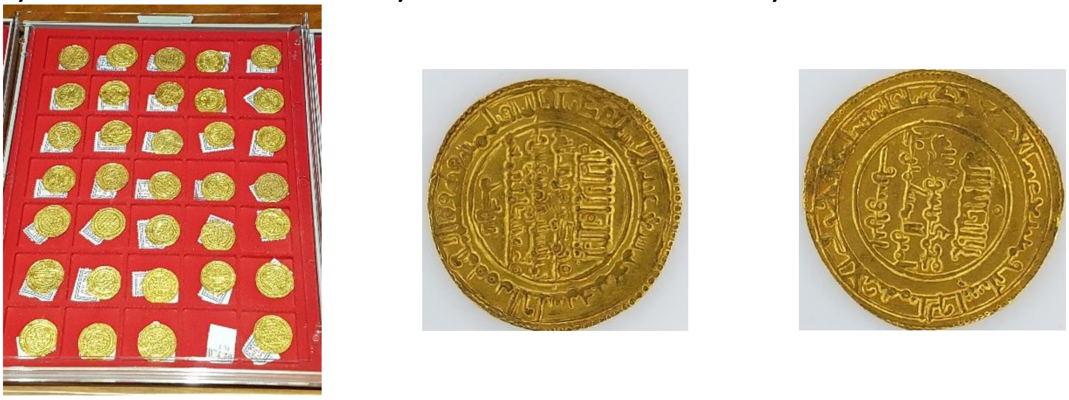

Figure S1: Overview of some of the investigated coins (**a**), and some zoomed-in coins B2M06 (**b**), and B2M09 (**c**).

IMAGE CAPTURE, CROPPING, SEGMENTATION AND CAMERA CHARACTERIZATION

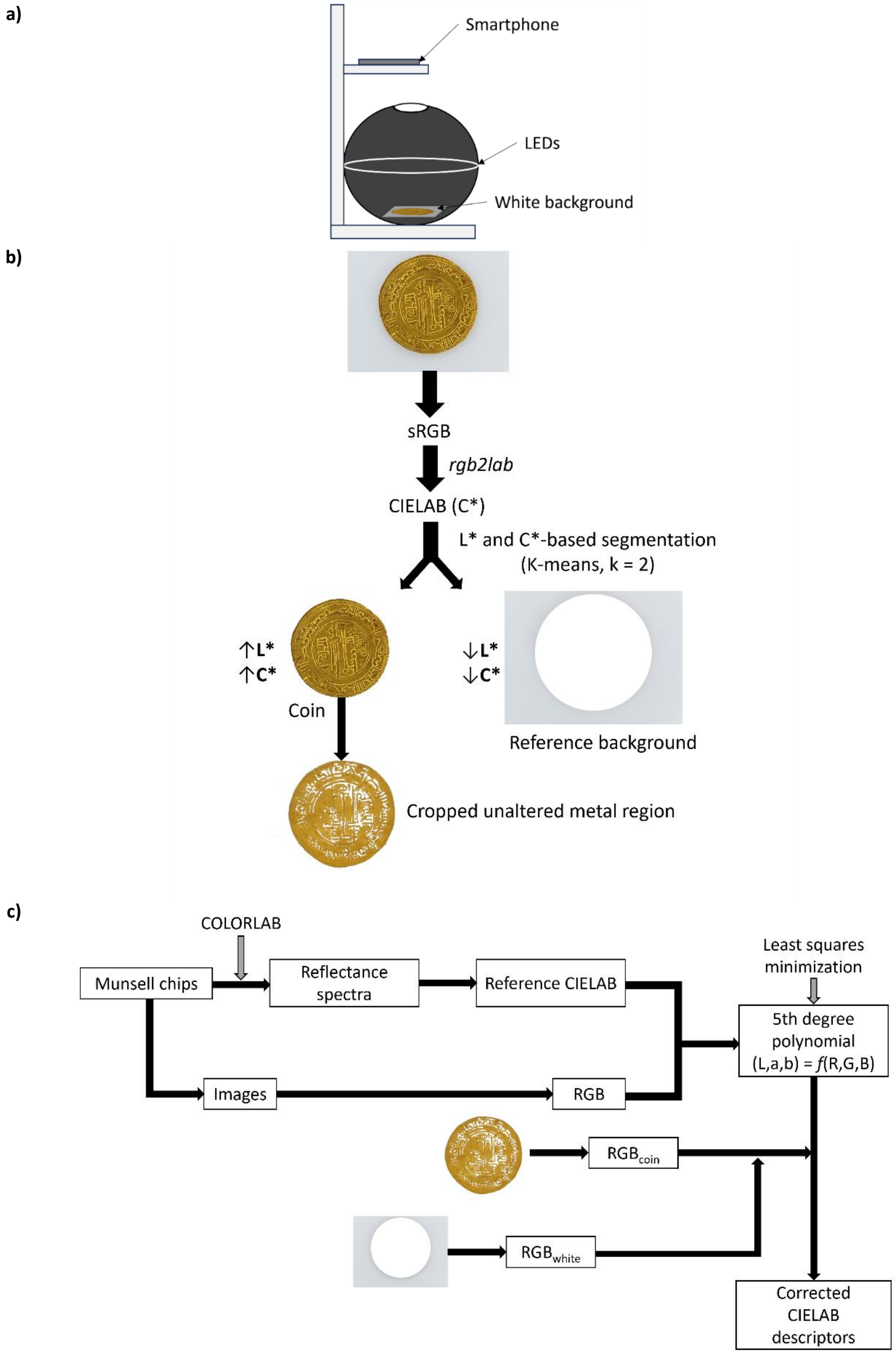

Figure S2: Smartphone-based setup (**a**), coin cropping (**b**) and smartphone characterization (**c**) steps proposed for gold coinage colorimetric investigations.

Table S2: Metallic content of the samples under investigation (mass percentage).

Sample	% Au	% Ag	% Cu	% Pt
B1M09	93.36 ± 0.11	1.53 ± 0.04	0.130 ± 0.017	<LOD
B1M10	91.79 ± 0.03	2.95 ± 0.11	0.233 ± 0.025	<LOD
B1M11	93.33 ± 0.13	1.29 ± 0.04	0.148 ± 0.010	<LOD
B1M12	90.18 ± 0.06	4.69 ± 0.11	0.305 ± 0.011	<LOD
B2M01	91.36 ± 0.18	3.43 ± 0.07	0.211 ± 0.011	0.21 ± 0.12
B2M02	93.09 ± 0.15	1.732 ± 0.019	0.116 ± 0.011	0.36 ± 0.18
B2M03	90.66 ± 0.14	4.21 ± 0.09	0.194 ± 0.006	0.28 ± 0.14
B2M04	90.77 ± 0.15	3.67 ± 0.08	0.239 ± 0.011	0.27 ± 0.16
B2M05	93.19 ± 0.10	1.74 ± 0.07	0.129 ± 0.006	<LOD
B2M06	89.32 ± 0.13	5.47 ± 0.07	0.35 ± 0.04	<LOD
B2M07	92.00 ± 0.17	2.86 ± 0.08	0.128 ± 0.005	<LOD
B2M08	89.45 ± 0.18	5.31 ± 0.10	0.393 ± 0.017	<LOD
B2M09	86.24 ± 0.25	8.48 ± 0.18	0.39 ± 0.03	<LOD
B2M10	91.46 ± 0.12	3.48 ± 0.07	0.207 ± 0.006	<LOD
B2M11	91.03 ± 0.17	3.73 ± 0.10	0.323 ± 0.013	<LOD
B2M12	90.13 ± 0.13	4.58 ± 0.09	0.330 ± 0.012	<LOD
B2M13	93.40 ± 0.14	1.59 ± 0.05	0.146 ± 0.006	<LOD
B2M14	92.49 ± 0.10	2.45 ± 0.08	0.153 ± 0.007	0.28 ± 0.14
B2M15	90.58 ± 0.08	4.11 ± 0.03	0.345 ± 0.011	<LOD
B2M16	90.2 ± 0.3	4.23 ± 0.10	0.293 ± 0.011	<LOD
B2M17	91.85 ± 0.18	3.11 ± 0.03	0.210 ± 0.016	<LOD
B2M18	89.8 ± 0.4	4.8 ± 0.3	0.299 ± 0.025	0.20 ± 0.10
B2M19	91.89 ± 0.20	2.91 ± 0.18	0.160 ± 0.021	<LOD
B2M20	92.1 ± 0.3	2.75 ± 0.24	0.125 ± 0.010	<LOD
B2M21	84.00 ± 0.22	10.7 ± 0.3	0.49 ± 0.03	0.24 ± 0.12
B2M22	84.8 ± 1.4	9.6 ± 1.1	1.0 ± 0.3	0.20 ± 0.11
B2M24	93.45 ± 0.13	1.42 ± 0.05	0.124 ± 0.009	<LOD
B2M25	90.60 ± 0.09	4.350 ± 0.016	0.240 ± 0.010	0.43 ± 0.21
B2M26	88.43 ± 0.19	6.35 ± 0.10	0.409 ± 0.012	0.21 ± 0.10
B2M27	89.58 ± 0.04	5.40 ± 0.10	0.317 ± 0.013	<LOD
B2M28	85.94 ± 0.15	8.49 ± 0.09	0.48 ± 0.05	0.20 ± 0.10
B2M29	93.08 ± 0.10	1.79 ± 0.03	0.144 ± 0.013	<LOD
B2M30	88.38 ± 0.21	6.24 ± 0.06	0.59 ± 0.11	0.27 ± 0.14
B2M31	89.92 ± 0.06	4.86 ± 0.04	0.348 ± 0.021	0.28 ± 0.14
B2M32	93.24 ± 0.08	1.67 ± 0.10	0.098 ± 0.021	<LOD
B2M33	90.14 ± 0.32	4.61 ± 0.12	0.155 ± 0.016	0.20 ± 0.09
B2M34	90.38 ± 0.11	4.46 ± 0.03	0.296 ± 0.015	0.29 ± 0.14
B2M35	90.16 ± 0.06	4.36 ± 0.03	0.336 ± 0.011	<LOD
B3M01	92.20 ± 0.16	2.519 ± 0.012	0.273 ± 0.024	<LOD
B3M02	89.46 ± 0.03	5.47 ± 0.11	0.178 ± 0.007	0.20 ± 0.10
B3M03	87.3 ± 0.5	7.5 ± 0.6	0.44 ± 0.07	<LOD
B3M04	89.2 ± 0.3	5.69 ± 0.15	0.35 ± 0.03	<LOD
B3M05	90.79 ± 0.14	4.00 ± 0.03	0.334 ± 0.014	<LOD
B3M06	92.40 ± 0.21	2.4 ± 0.3	0.153 ± 0.011	<LOD
B3M08	88.7 ± 0.3	5.84 ± 0.11	0.537 ± 0.023	<LOD
B3M09	93.06 ± 0.10	1.803 ± 0.016	0.152 ± 0.011	<LOD
B3M10	90.03 ± 0.13	4.58 ± 0.08	0.224 ± 0.012	<LOD
B3M11.1	88.75 ± 0.17	5.86 ± 0.13	0.365 ± 0.023	<LOD
B3M11.2	93.41 ± 0.15	1.54 ± 0.07	0.121 ± 0.013	<LOD
B3M12	89.9 ± 0.3	4.98 ± 0.06	0.185 ± 0.011	0.22 ± 0.11
B3M13	92.5 ± 0.4	2.4 ± 0.3	0.155 ± 0.009	<LOD
B3M14	89.74 ± 0.09	5.018 ± 0.009	0.385 ± 0.017	0.26 ± 0.13
B3M15	92.0 ± 0.8	1.60 ± 0.04	0.233 ± 0.011	0.22 ± 0.13
B3M16	81.3 ± 0.5	12.8 ± 0.4	1.20 ± 0.14	<LOD
B3M17	88.91 ± 0.12	5.89 ± 0.23	0.230 ± 0.019	<LOD
B3M18	91.01 ± 0.18	3.51 ± 0.09	0.217 ± 0.015	<LOD
B3M19	89.89 ± 0.06	4.844 ± 0.024	0.458 ± 0.013	<LOD
B3M20	88.4 ± 0.5	6.0 ± 0.3	0.346 ± 0.021	<LOD

a)

b)

c)

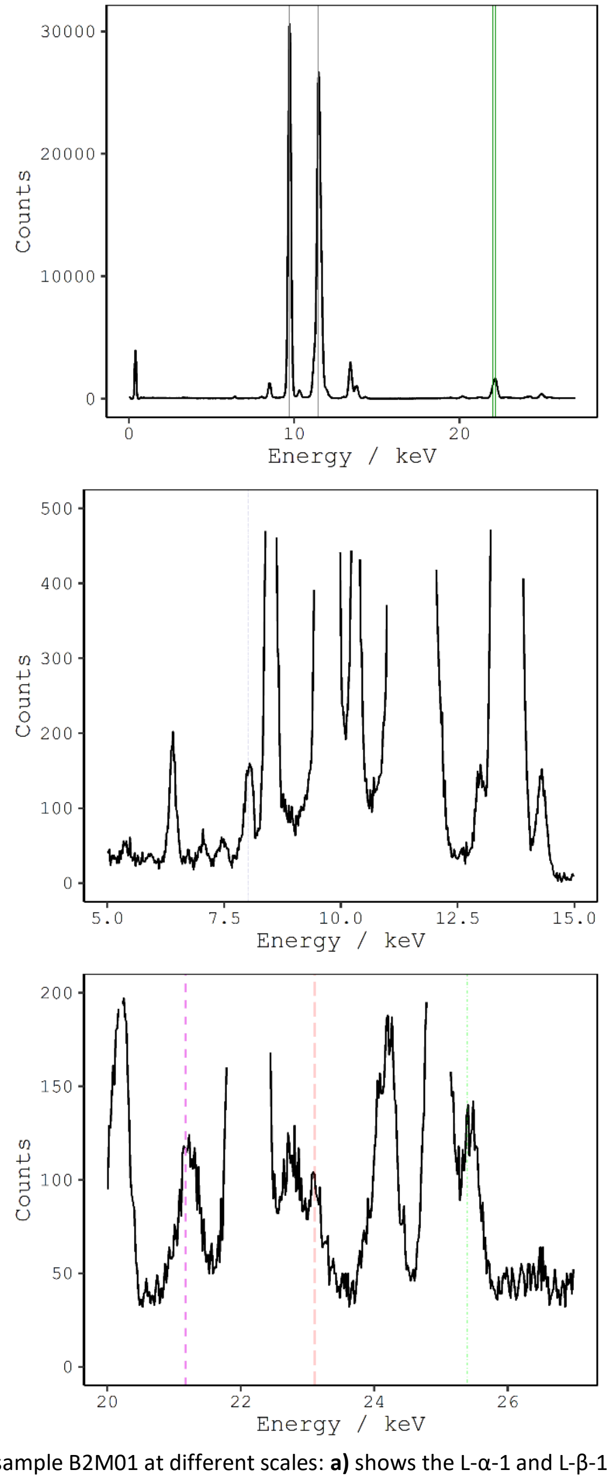

Figure S3: XRF spectrum of sample B2M01 at different scales: **a)** shows the L-α-1 and L-β-1 bands of Au (9.7 and 11.4 keV, respectively) in grey, and the K-α-2 and K-α-1 of Au in green (21.9 and 22.1 keV). **b)** K-α-1 and K-α-2 of Cu at 8.04 and 8.02 keV. **c)** Pd(K-α-1): 21.1 keV; Cd(K-α-1): 23.1 keV; Sn(K-α-1): 25.3 keV.

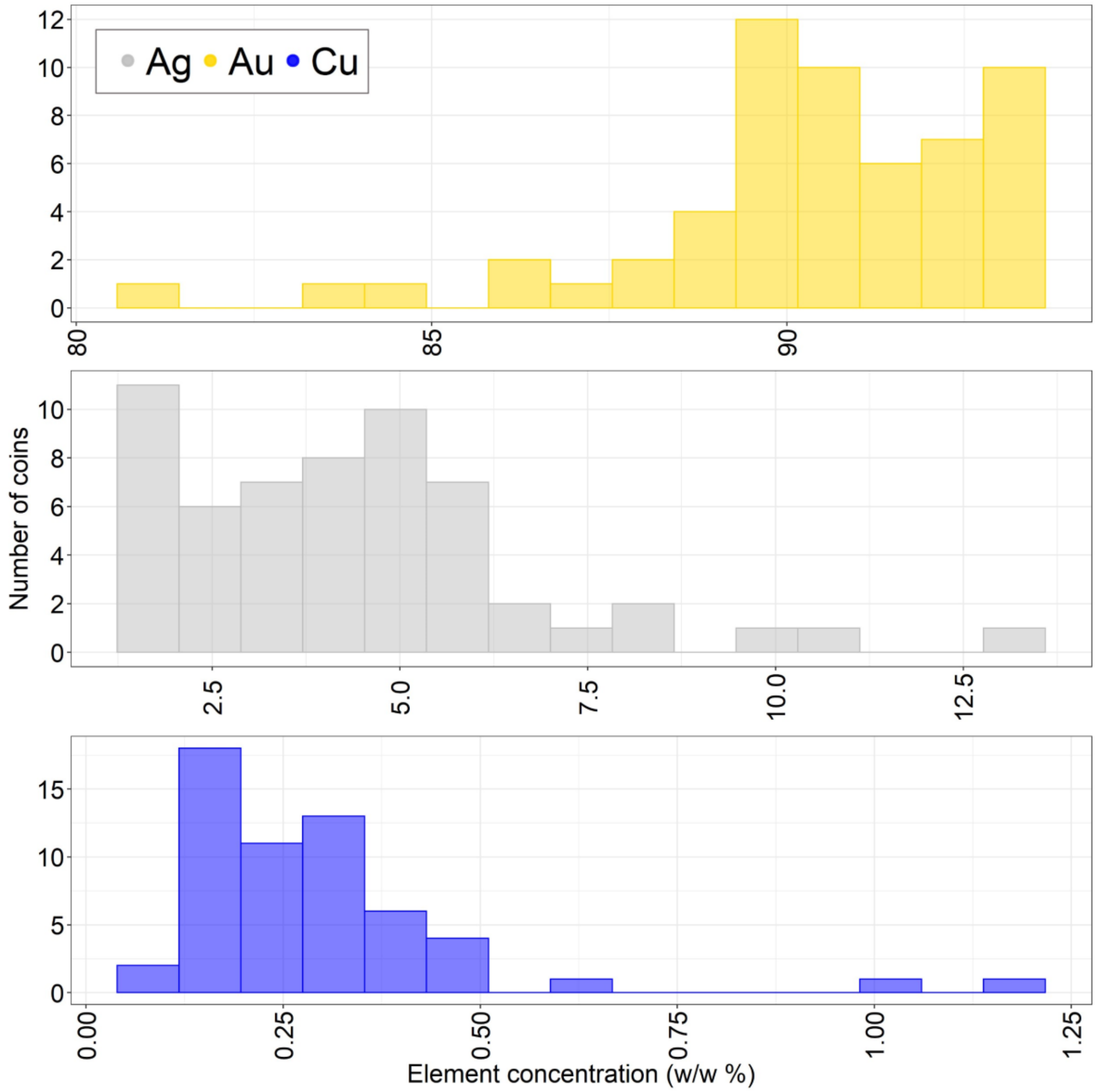

Figure S4: Distribution of the values for Au, Ag and Cu for the Islamic coins under investigation.

Access to dataset

Research data is available at https://doi.org/10.5281/zenodo.10382244. It contains the elemental profile of the coins used for their description in the manuscript, along with the physical properties (size and mass) and the colorimetric descriptors. Also, the CIELAB data obtained both by the imaging method and the spectroradiometer is presented.
